# Supplementary figures and images for: Seamless trials in oncology: A cross-sectional analysis of characteristics and reporting
Source: PLoS One. 2024 Dec 3;19(12):e0312797. doi: 10.1371/journal.pone.0312797 (PMC11614237; doi:10.1371/journal.pone.0312797)

**
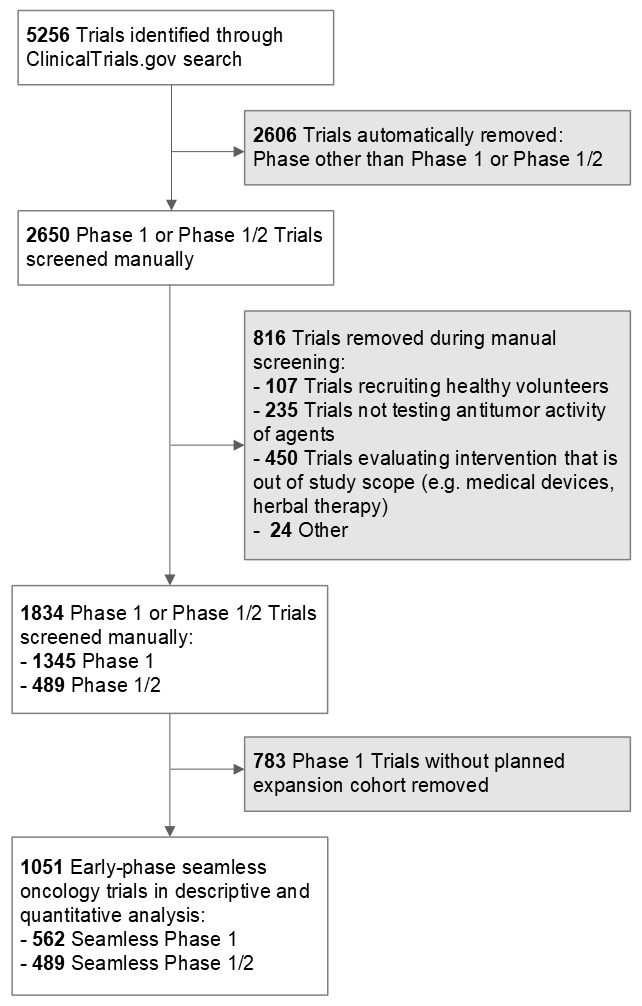
**

**S1 Fig. Flow diagram for identification of seamless early-phase oncology trials**

Supplement: S1 Fig — (DOCX) [file pone.0312797.s001.docx]
