# Supplementary material for: Seamless trials in oncology: A cross-sectional analysis of characteristics and reporting
Source: PLoS One. 2024 Dec 3;19(12):e0312797. doi: 10.1371/journal.pone.0312797 (PMC11614237; doi:10.1371/journal.pone.0312797)
Supplement: S2 Fig — (DOCX) [file pone.0312797.s002.docx]

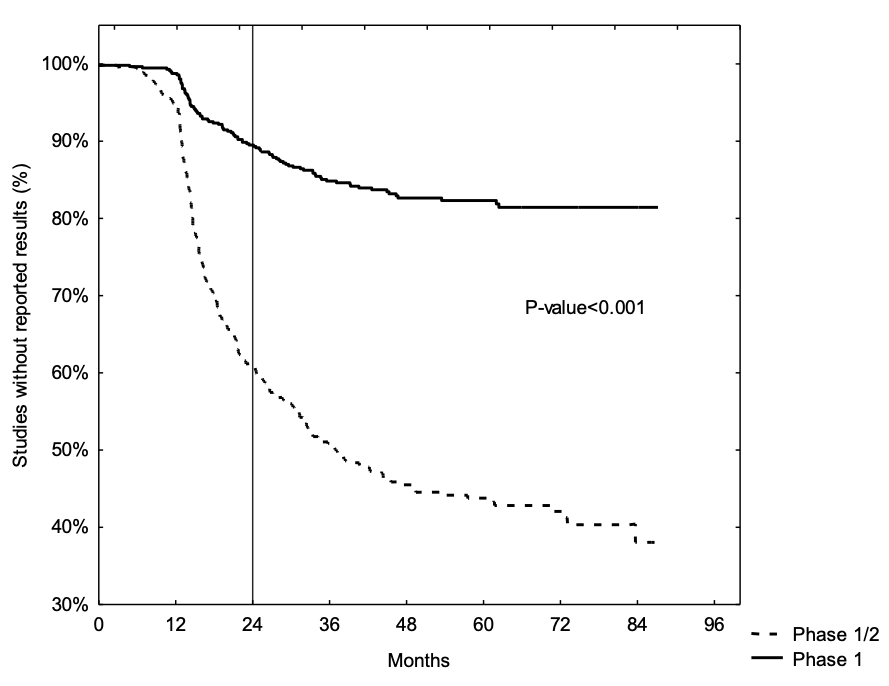
**S2 Figure. Kaplan-Meier curve for the cumulative probability of not reporting clinical trial results over time – comparison between seamless Phase 1 and Phase 1/2**
