# Supplementary material for: Seamless trials in oncology: A cross-sectional analysis of characteristics and reporting
Source: PLoS One. 2024 Dec 3;19(12):e0312797. doi: 10.1371/journal.pone.0312797 (PMC11614237; doi:10.1371/journal.pone.0312797)
Supplement: S1 Table — (DOCX) [file pone.0312797.s004.docx]

**S1 Table. STROBE Statement - checklist of items that should be included in reports of cross-sectional studies**

|  | Item No. | Recommendation |  | Relevant text from manuscript |
| --- | --- | --- | --- | --- |
| **Title and abstract** | 1 | (*a*) Indicate the study’s design with a commonly used term in the title or the abstract |  | Title, Abstract |
|  |  | (*b*) Provide in the abstract an informative and balanced summary of what was done and what was found |  | Abstract |
| Introduction | | | |  |
| Background/rationale | 2 | Explain the scientific background and rationale for the investigation being reported |  | Introduction: Paragraphs 1-4 |
| Objectives | 3 | State specific objectives, including any prespecified hypotheses |  | Introduction: Paragraph 5 |
| Methods | | | |  |
| Study design | 4 | Present key elements of study design early in the paper |  | Methods section |
| Setting | 5 | Describe the setting, locations, and relevant dates, including periods of recruitment, exposure, follow-up, and data collection |  | Methods: Sample and trial selection - Paragraph 1 |
| Participants | 6 | Give the eligibility criteria, and the sources and methods of selection of participants |  | Methods: Sample and trial selection - Paragraph 2 |
| Variables | 7 | Clearly define all outcomes, exposures, predictors, potential confounders, and effect modifiers. Give diagnostic criteria, if applicable |  | Methods: Data collection  S3 Table |
| Data sources/ measurement | 8 | For each variable of interest, give sources of data and details of methods of assessment (measurement). Describe comparability of assessment methods if there is more than one group |  | Methods:  Data collection |
| Bias | 9 | Describe any efforts to address potential sources of bias |  | Methods: Sample and trial selection - Paragraph 3  Data collection - Paragraph 1 |
| Study size | 10 | Explain how the study size was arrived at |  | Fig 1  S1 Fig |
| Quantitative variables | 11 | Explain how quantitative variables were handled in the analyses. If applicable, describe which groupings were chosen and why |  | Methods: Statistical analysis |
| Statistical methods | 12 | (*a*) Describe all statistical methods, including those used to control for confounding |  | Methods: Statistical analysis and tools |
|  |  | (*b*) Describe any methods used to examine subgroups and interactions |  | Methods: Statistical analysis and tools - Paragraph 2 |
|  |  | (*c*) Explain how missing data were addressed |  | Methods: Statistical analysis and tools - Paragraph 2 |
|  |  | (*d*) If applicable, describe analytical methods taking account of sampling strategy |  | Not applicable |
|  |  | (*e*) Describe any sensitivity analyses |  | Not applicable |
| **Results** |  |  |  |  |
| Participants | 13 | (a) Report numbers of individuals at each stage of study—eg numbers potentially eligible, examined for eligibility, confirmed eligible, included in the study, completing follow-up, and analysed |  | S1 Fig  Results: Trial characteristics |
|  |  | (b) Give reasons for non-participation at each stage |  | S1 Fig |
|  |  | (c) Consider use of a flow diagram |  | S1 Fig |
| Descriptive data | 14 | (a) Give characteristics of study participants (eg demographic, clinical, social) and information on exposures and potential confounders |  | Results: Trial characteristics |
|  |  | (b) Indicate number of participants with missing data for each variable of interest |  | Table 1 |
| Outcome data | 15 | *Cross-sectional study—*Report numbers of outcome events or summary measures |  | Results,  Table 1  Table 2  Table 3  S4-8 Tables |
| Main results | 16 | (*a*) Give unadjusted estimates and, if applicable, confounder-adjusted estimates and their precision (eg, 95% confidence interval). Make clear which confounders were adjusted for and why they were included |  | Table 1  Table 2  Table 3 |
|  |  | (*b*) Report category boundaries when continuous variables were categorized |  | Not applicable |
|  |  | (*c*) If relevant, consider translating estimates of relative risk into absolute risk for a meaningful time period |  | Not applicable |
| Other analyses | 17 | Report other analyses done—eg analyses of subgroups and interactions, and sensitivity analyses |  | Results section |
| **Discussion** |  |  |  |  |
| Key results | 18 | Summarise key results with reference to study objectives |  | Discussion – Paragraph 2  Conclusions section |
| Limitations | 19 | Discuss limitations of the study, taking into account sources of potential bias or imprecision. Discuss both direction and magnitude of any potential bias |  | Limitations section |
| Interpretation | 20 | Give a cautious overall interpretation of results considering objectives, limitations, multiplicity of analyses, results from similar studies, and other relevant evidence |  | Discussion section |
| Generalisability | 21 | Discuss the generalisability (external validity) of the study results |  | Discussion section |
| **Other information** |  |  |  |  |
| Funding | 22 | Give the source of funding and the role of the funders for the present study and, if applicable, for the original study on which the present article is based |  | Funding section |
