# Supplementary material for: Seamless trials in oncology: A cross-sectional analysis of characteristics and reporting
Source: PLoS One. 2024 Dec 3;19(12):e0312797. doi: 10.1371/journal.pone.0312797 (PMC11614237; doi:10.1371/journal.pone.0312797)
Supplement: S2 Table — (DOCX) [file pone.0312797.s005.docx]

**S2 Table. ClinicalTrials.gov search parameters**

| **Criterion** | **Search term(s)** |
| --- | --- |
| Condition or disease | Cancer |
| Study Type | Interventional studies (Clinical Trials) |
| Status | Completed |
| Phase | Phase 1 OR Phase 2 |
| Primary Completion Date | 01/01/2016 – 12/31/2020 |

We used the search tool available on the website: <https://classic.clinicaltrials.gov/ct2/search/advanced>
