# Supplementary material for: Seamless trials in oncology: A cross-sectional analysis of characteristics and reporting
Source: PLoS One. 2024 Dec 3;19(12):e0312797. doi: 10.1371/journal.pone.0312797 (PMC11614237; doi:10.1371/journal.pone.0312797)
Supplement: S3 Table — (DOCX) [file pone.0312797.s006.docx]

**S3 Table. Details on data categorization for trial characteristics**

| **Characteristic** | **Categories** | **Description** |
| --- | --- | --- |
| Enrollment | Not applicable | Refers to the number of patients enrolled based on the information reported in Study Details (reported as “Actual Enrollment" or "Estimated Enrollment"). |
| Study start date | Not applicable | Refers to data reported in “Study Start”. |
| Primary completion date | Not applicable | Refers to data reported in “Primary Completion”. |
| Funder type | -Industry  -Non-industry  -Partially industry | Assessment based on “Funder Type” section.  Non-industry refers to all terms other than "Industry".  If more than one type of funding was reported on ClinicalTrials.gov, and one of them was industry, we classified the trial as partially funded by industry. |
| Study population age | -Pediatric  -Adults  -Both | Pediatric refers to age birth to 17 years and adult refers to age ≥ 18 years. Assessment based on “Ages eligible for study”. |
| Type of intervention | -Cytotoxic therapy  -Targeted therapy  -Immunotherapy  -Other  -Mixed | As defined by the National Cancer Institute:  “*Cytotoxic therapy* refers to anticancer drugs that kill cells, especially cancer cells. These agents may stop cancer cells from dividing and growing and may cause tumors to shrink in size. Examples are alkylating agents, antimetabolites, topoisomerase inhibitors.”  “*Targeted therapy* is a type of cancer treatment that targets proteins that control how cancer cells grow, divide, and spread. Most targeted therapies are either small-molecule drugs or monoclonal antibodies. Examples are angiogenesis inhibitors and proteasome inhibitors.”  “*Immunotherapy* is a type of cancer treatment that helps immune system fight cancer. Several types of immunotherapies are used to treat cancer including immune checkpoint inhibitors, T-cell transfer therapy, monoclonal antibodies, treatment vaccines, immune system modulators.”  Following the method proposed in other study^[[1]](#footnote-1)^, during the classification of the intervention type, we used a hierarchical model.  Drugs with properties of more than one class were classified according to their most innovative mechanism.  Immunotherapy was considered as the most innovative therapy, followed by targeted and cytotoxic therapy.  Drugs that did not fit into any of these three categories were listed as "other" (e.g. hormone therapy, gene therapy, antifungals).  If the clinical trial tested two or more drugs that could be classified as different types of therapy, e.g., immunotherapy + cytotoxic therapy; immunotherapy + other; targeted therapy + other, etc., we chose "mixed".  If the trial tested two or more drugs of the same type, e.g. two targeted therapy drugs, we classified it as that specific therapy, i.e. targeted therapy. |
| Number of drugs evaluated in the study | -Single agent  -Multiple agents | “Single agent” refers to one agent being evaluated in the entire study. "Multiple agent" is when several different agents are under evaluation. |
| Type of cancer | -Solid  -Hematological  -Both  -N/R | As defined by the National Cancer Institute:  -Solid tumor: An abnormal mass of tissue that usually does not contain cysts or liquid areas. Solid tumors may be benign (not cancer), or malignant (cancer). Different types of solid tumors are named for the type of cells that form them (based on National Cancer Institute’s definition). Examples of solid tumors: breast cancer, prostate cancer, lung cancer.  -Hematologic cancer: Cancer that begins in blood-forming tissue, such as the bone marrow, or in the cells of the immune system. Examples of hematologic cancer are leukemia, lymphoma, and multiple myeloma. |
| Number of cancer types | -Single  -Multiple | "Single" if one cancer type was evaluated.  "Multiple" if more than one cancer type was evaluated.  In case it was not reported we selected "multiple". |
| Masking | -None (open label)  -Single blind  -At least double-blind  -N/R | Assessment based on "Design details" section and description of the study. "At least double-blind" refers to double-blind, triple-blind, and quadruple-blind. N/R - if not reported. |
| Randomization | -Randomized  -Non-randomized  -Partially randomized  N/R | Assessment based on the "Design details" (see “Allocation”) section and Study Description and Arms. "Partially randomized" - if one of the study arms was randomized and the other was not.  N/R – if no information was reported. |
| Interventional model | -Single group  -Parallel  -Sequential  -Factorial  -Crossover  -N/R | Refers to “Interventional Model” in “Design details” section. |
| Number of trial’s sites | -Single-site  -Multi-site  -N/R | If only one location was reported – single-site. If more than one – multi-site. Sometimes this information was not present – N/R. |
| Recruitment regions #1 | -North America  -Europe  -Asia  -South America  -Africa  -Australia  -Mixed  -N/R | Refers to the continents where the trials were conducted. "Mixed" if trials were conducted on different continents. |
| Recruitment regions #2 | -US  -Non-US  -Multicenter including US  -N/R | If all the study sites were in the United States (US) - "US". "Non-US" if all sites were outside the US. "Multicenter including US" if some sites were in the US and some were outside the US. If no information - N/R. |

1. Hutchinson N, Carlisle B, Doussau A, et al. Patient Participation in Clinical Trials of Oncology Drugs and Biologics Preceding Approval by the US Food and Drug Administration. JAMA Network Open 2021;4(5):e2110456-e56. doi: 10.1001/jamanetworkopen.2021.10456 [↑](#footnote-ref-1)
