# Supplementary material for: Seamless trials in oncology: A cross-sectional analysis of characteristics and reporting
Source: PLoS One. 2024 Dec 3;19(12):e0312797. doi: 10.1371/journal.pone.0312797 (PMC11614237; doi:10.1371/journal.pone.0312797)
Supplement: S4 Table — (DOCX) [file pone.0312797.s007.docx]

**S4 Table. Characteristics of a seamless Phase 1 study and a Phase 1/2 study**

| **Characteristic** | **Phase 1**  **N=562 (100%)** | **Phase 1/2**  **N=489 (100%)** |
| --- | --- | --- |
| **Enrolled participants** |  |  |
| 1-50 | 317 (56.4%) | 277 (56.6%) |
| 51-100 | 139 (24.7%) | 107 (21.9%) |
| 101-150 | 51 (9.1%) | 54 (11.0%) |
| 151-200 | 29 (5.2%) | 19 (3.9%) |
| >200 | 26 (4.6%) | 32 (6.5%) |
| **Study start date** |  |  |
| ≤2010 | 34 (6.0%) | 57 (11.7%) |
| 2011-2012 | 98 (17.4%) | 90 (18.4%) |
| 2013-2014 | 194 (34.5%) | 131 (26.8%) |
| 2015-2016 | 158 (28.1%) | 143 (29.2%) |
| 2017-2018 | 74 (13.2%) | 64 (13.1%) |
| 2019-2020 | 4 (0.7%) | 4 (0.8%) |
| **Primary completion date** |  |  |
| 2016 | 100 (17.8%) | 90 (18.4%) |
| 2017 | 118 (21.0%) | 87 (17.8%) |
| 2018 | 98 (17.4%) | 95 (19.4%) |
| 2019 | 131 (23.3%) | 108 (22.1%) |
| 2020 | 115 (20.5%) | 109 (22.3%) |
| **Funder type** |  |  |
| Industry | 375 (66.7%) | 221 (45.2%) |
| Non-industry | 84 (14.9%) | 154 (31.5%) |
| Partially-industry | 103 (18.3%) | 114 (23.3%) |
| **Study population age** |  |  |
| Adults | 537 (95.6%) | 450 (92.0%) |
| Pediatrics | 0 (0.0%%) | 3 (0.6%) |
| Both | 25 (4.4%%) | 36 (7.4%) |
| **Number of drugs evaluated in the study** |  |  |
| Single agent | 245 (43.6%) | 152 (31.1%) |
| Multiple agents | 317 (56.4%) | 337 (68.9%) |
| **Type of cancer** |  |  |
| Solid | 418 (74.4%) | 334 (68.3%) |
| Hematological | 108 (19.2%) | 142 (29.0%) |
| Both | 33 (5.9%) | 13 (2.7%) |
| **Number of cancer types** |  |  |
| Single | 197 (35.1%) | 324 (66.3%) |
| Multiple | 365 (64.9%) | 165 (33.7%) |
| **Masking** |  |  |
| Open label | 561 (99.8%) | 471 (96.3%) |
| Single blind | 0 (0.0%) | 1 (0.2%) |
| At least double-blind | 0 (0.0%) | 15 (3.1%) |
| **Randomization** |  |  |
| Non-randomized | 531 (94.5%) | 391 (80.0%) |
| Partially randomized | 6 (1.1%) | 50 (10.2%) |
| Randomized | 16 (2.8%) | 39 (8.0%) |
| **Number of trial's sites** |  |  |
| Single-site | 139 (24.7%) | 168 (34.4%) |
| Multi-site | 414 (73.7%) | 319 (65.2%) |
| **Location** |  |  |
| United States (US) | 255 (45.4%) | 221 (45.2%) |
| Multicenter including US | 173 (30.8%) | 93 (19.0%) |
| Non-US | 125 (22.2%) | 173 (35.4%) |
