# Supplementary material for: Seamless trials in oncology: A cross-sectional analysis of characteristics and reporting
Source: PLoS One. 2024 Dec 3;19(12):e0312797. doi: 10.1371/journal.pone.0312797 (PMC11614237; doi:10.1371/journal.pone.0312797)
Supplement: S5 Table — (DOCX) [file pone.0312797.s008.docx]

**S5 Table.** **Type of cancer**

| **Type of cancer** | **Trials with single cancer type, N=521 (100%)** | **Trials with multiple cancer types, N=530 (100%)** | **All trials**  **N=1051 (100%)** |
| --- | --- | --- | --- |
| Lung Cancer | 62 (11.9%) | 134 (25.3%) | 196 (18.6%) |
| Breast Cancer | 55 (10.6%) | 113 (21.3%) | 168 (16.0%) |
| Lymphoma (All types) | 66 (12.7%) | 80 (15.1%) | 146 (13.9%) |
| Leukemia (All Types) | 49 (9.4%) | 82 (15.5%) | 131 (12.5%) |
| Ovarian Cancer (or Fallopian Tube or Peritoneal Cancer) | 9 (1.7%) | 103 (19.4%) | 112 (10.7%) |
| Colon and Rectal Cancer (Combined) | 29 (5.6%) | 77 (14.5%) | 106 (10.1%) |
| Gastric Cancer (or Gastroesophageal Cancer or Gastrointestinal Cancer) | 18 (3.5%) | 77 (14.5%) | 95 (9.0%) |
| Pancreatic Cancer | 33 (6.3%) | 50 (9.4%) | 83 (7.9%) |
| Melanoma | 22 (4.2%) | 56 (10.6%) | 78 (7.4%) |
| Kidney Cancer | 17 (3.3%) | 46 (8.7%) | 63 (6.0%) |
| Prostate Cancer | 28 (5.4%) | 35 (6.6%) | 63 (6.0%) |
| Head and neck cancer (including Tongue Cancer or Nasopharyngeal Cancer) | 9 (1.7%) | 52 (9.8%) | 61 (5.8%) |
| Multiple Myeloma | 40 (7.7%) | 18 (3.4%) | 58 (5.5%) |
| Bladder Cancer | 6 (1.2%) | 45 (8.5%) | 51 (4.9%) |
| Liver Cancer | 23 (4.4%) | 28 (5.3%) | 51 (4.9%) |
| Endometrial Cancer or Cervical Cancer | 4 (0.8%) | 39 (7.4%) | 43 (4.1%) |
| Myelodysplastic Syndrome | 7 (1.3%) | 30 (5.7%) | 37 (3.5%) |
| Brain cancer (including glioma and glioblastoma) | 15 (2.9%) | 21 (4.0%) | 36 (3.4%) |
| Sarcoma | 8 (1.5%) | 26 (4.9%) | 34 (3.2%) |
| Cholangiocarcinoma (Biliary Tract Cancer) | 5 (1.0%) | 14 (2.6%) | 19 (1.8%) |
| Skin cancer (other than melanoma) | 7 (1.3%) | 11 (2.1%) | 18 (1.7%) |
| Thyroid Cancer | 2 (0.4%) | 7 (1.3%) | 9 (0.9%) |
| Other^a^ | 7 (1.3%) | 243 (45.8%) | 250 (23.8%) |
| N/R | N/A | 109 (20.6%) | 109 (10.4%) |

^a^In many cases the study description record did not report on all cancer types considered. The term "other" was often used instead of or in addition to a specific cancer type.

## N/A: not applicable. N/R: not reported.
