# Supplementary material for: Seamless trials in oncology: A cross-sectional analysis of characteristics and reporting
Source: PLoS One. 2024 Dec 3;19(12):e0312797. doi: 10.1371/journal.pone.0312797 (PMC11614237; doi:10.1371/journal.pone.0312797)
Supplement: S6 Table — (DOCX) [file pone.0312797.s009.docx]

**S6 Table. Details on studies with an expansion cohort directly mentioned**

| **Characteristics** | **All trials,**  **N=1051 (100%)** | **Phase 1,**  **N=562 (100%)** | **Phase 1/2,**  **N=489 (100%)** |
| --- | --- | --- | --- |
| **Expansion cohort directly mentioned** | 665 (63.3%) | 518 (92.2%) | 147 (30.1%) |
| **In which section was it reported?** | | | |
| 1. Title (short or official) | 92/665 (13.8%) | 81/518 (15.6%) | 11/147 (7.5%) |
| 2. Brief and Detailed Description  (excluding title) | 376//665 (56.5%) | 277/518 (53.5%) | 99/147 (67.3%) |
| 3. Arms and Interventions | 108/665 (16.2%) | 77/518 (14.9%) | 31/147 (21.1%) |
| 4. Participation Criteria | 72/665 (10.8%) | 67/518 (12.9%) | 5/147 (3.4%) |
| 5. Other | 17/665 (2.6%) | 16/518 (3.1%) | 1/147 (0.7%) |
