# Supplementary material for: Seamless trials in oncology: A cross-sectional analysis of characteristics and reporting
Source: PLoS One. 2024 Dec 3;19(12):e0312797. doi: 10.1371/journal.pone.0312797 (PMC11614237; doi:10.1371/journal.pone.0312797)
Supplement: S7 Table — (DOCX) [file pone.0312797.s010.docx]

**S7 Table. Characteristic of the endpoints**

| **Type of outcome** | **All trials,**  **N=1051 (%^)a^** |  | **Seamless Phase 1,**  **N=562 (100%)^a^** | **Phase 1/2, N=489 (100%)^a^** | **P-value** |
| --- | --- | --- | --- | --- | --- |
| **MTD or DLTs** | **747 (71.1%)** |  | **446 (79.4%)** | **301 (61.6%)** | **<0.001** |
| *primary* | 699 (93.6%) |  | 412 (92.4%) | 287 (95.3%) | 0.028 |
| *secondary* | 19 (2.5%) |  | 17 (3.8%) | 2 (0.7%) |  |
| *both* | 29 (3.9%) |  | 17 (3.8%) | 12 (4.0%) |  |
| **RP2D** | **168 (16.0%)** |  | **106 (18.9%)** | **62 (12.7%)** | **0.006** |
| *primary* | 145 (86.3%) |  | 91 (85.8%) | 54 (87.1%) | 1 |
| *secondary* | 22 (13.1%) |  | 14 (13.2%) | 8 (12.9%) |  |
| *both* | 1 (0.6%) |  | 1 (0.9%) | 0 (0.0%) |  |
| **PFS** | **518 (49.3%)** |  | **250 (44.5%)** | **268 (54.8%)** | **<0.001** |
| *primary* | 60 (11.6%) |  | 8 (3.2%) | 52 (19.4%) | <0.001 |
| *secondary* | 429 (82.8%) |  | 236 (94.4%) | 193 (72.0%) |  |
| *both* | 29 (5.6%) |  | 6 (2.4%) | 23 (8.6%) |  |
| **OS** | **388 (36.9%)** |  | **147 (26.2%)** | **241 (49.3%)** | **<0.001** |
| *primary* | 22 (5.7%) |  | 2 (1.4%) | 20 (8.3%) | 0.005 |
| *secondary* | 360 (92.8%) |  | 144 (98.0%) | 216 (89.6%) |  |
| *both* | 6 (1.5%) |  | 1 (0.7%) | 5 (2.1%) |  |
| **RR** | **856 (81.4%)** |  | **450 (80.1%)** | **406 (83.0%)** | **0.219** |
| *primary* | 152 (17.8%) |  | 35 (7.8%) | 117 (28.8%) | <0.001 |
| *secondary* | 567 (66.2%) |  | 374 (83.1%) | 193 (47.5%) |  |
| *both* | 137 (16.0%) |  | 41 (9.1%) | 96 (23.6%) |  |
| **TRAEs** | **142 (13.5%)** |  | **70 (12.5%)** | **72 (14.7%)** | **0.283** |
| *primary* | 77 (54.2%) |  | 42 (60.0%) | 35 (48.6%) | 0.290 |
| *secondary* | 58 (40.8%) |  | 26 (37.1%) | 32 (44.4%) |  |
| *both* | 7 (4.9%) |  | 2 (2.9%) | 5 (6.9%) |  |
| **Pharmacokinetics measures** | **559 (53.2%)** |  | **384 (68.3%)** | **175 (35.8%)** | **<0.001** |
| *primary* | 48 (8.6%) |  | 35 (9.1%) | 13 (7.4%) | 0.077 |
| *secondary* | 488 (87.3%) |  | 338 (88.0%) | 150 (85.7%) |  |
| *both* | 23 (4.1%) |  | 11 (2.9%) | 12 (6.9%) |  |

^a^Percentages for endpoint type (primary, secondary, both) refer to studies in which that endpoint occurred.

## AEs: adverse events. DLTs: dose-limiting toxicities. MTD: maximum tolerated dose. OS: overall survival. PFS: progression-free survival. RP2D: recommended phase 2 dose. RR: response rates. TRAEs: treatment-related AEs.
