# Supplementary material for: Seamless trials in oncology: A cross-sectional analysis of characteristics and reporting
Source: PLoS One. 2024 Dec 3;19(12):e0312797. doi: 10.1371/journal.pone.0312797 (PMC11614237; doi:10.1371/journal.pone.0312797)
Supplement: S8 Table — (DOCX) [file pone.0312797.s011.docx]

**S8 Table. Comparison of time to reporting results based on trial characteristics**

| **Characteristic** | **Median [months]** | **Q1-Q3** | **P-Value** |
| --- | --- | --- | --- |
| **Phase** |  |  |  |
| Phase 1 | 20 | (14 - 29) | 0.147 |
| Phase 1/2 | 17 | (14 - 26) |  |
| **Enrolled participants** |  |  |  |
| 1-50 | 19 | (14 - 28) | 0.617 |
| 51-100 | 16 | (14 - 29) |  |
| 101-150 | 17 | (14 - 25) |  |
| 151-200 | 18 | (14 - 26) |  |
| >200 | 19 | (14 - 28) |  |
| **Study start date** |  |  |  |
| ≤2010 | 17 | (14 - 30) | 0.003 |
| 2011-2012 | 22 | (14 - 33) |  |
| 2013-2014 | 19 | (14 - 28) |  |
| 2015-2016 | 15 | (14 - 22) |  |
| 2017-2018 | 16 | (13 - 21) |  |
| 2019-2020 | 17 | (14 - 30) |  |
| **Primary completion date** |  |  |  |
| 2016 | 24 | (14 - 42) | <0.001 |
| 2017 | 24 | (18 - 33) |  |
| 2018 | 20 | (14 - 27) |  |
| 2019 | 14 | (13 - 22) |  |
| 2020 | 15 | (13 - 17) |  |
| **Funder type** |  |  |  |
| Industry | 18 | (14 - 27) | 0.406 |
| Non-industry | 18 | (14 - 28) |  |
| Partially-industry | 16 | (13 - 24) |  |
| **Study population age** |  |  |  |
| Adults | 18 | (14 - 27) | 0.03 |
| Pediatrics | 12 | (7 - 13) |  |
| Both | 15 | (12 - 28) |  |
| **Number of drugs evaluated in the study** |  |  |  |
| Single agent | 19 | (14 - 27) | 0.168 |
| Multiple agents | 16 | (13 - 27) |  |
| **Type of cancer** |  |  |  |
| Solid | 18 | (14 - 28) | 0.281 |
| Hematological | 16 | (14 - 23) |  |
| Both | 23 | (13 - 39) |  |
| **Number of cancer types** |  |  |  |
| Single | 18 | (14 - 29) | 0.417 |
| Multiple | 16 | (14 - 25) |  |
| **Masking** |  |  |  |
| Open label | 17 | (14 - 27) | 0.079 |
| Single blind | 29 | (29 - 29) |  |
| At least double-blind | 26 | (18 - 37) |  |
| **Randomization** |  |  |  |
| Non-randomized | 17 | (14 - 27) | 0.460 |
| Partially randomized | 19 | (14 - 31) |  |
| Randomized | 15 | (13 - 22) |  |
| **Number of trial's sites** |  |  |  |
| Single-site | 17 | (13 - 28) | 0.436 |
| Multi-site | 18 | (14 - 27) |  |
| **Location** |  |  |  |
| United States (US) | 18 | (13 - 27) | 0.050 |
| Multicenter including US | 15 | (13 - 25) |  |
| Non-US | 21 | (15 - 30) |  |

Data presented for clinical trials with results reported.
