# Supplementary material for: Seamless trials in oncology: A cross-sectional analysis of characteristics and reporting
Source: PLoS One. 2024 Dec 3;19(12):e0312797. doi: 10.1371/journal.pone.0312797 (PMC11614237; doi:10.1371/journal.pone.0312797)
Supplement: Attachment — Submitted filename: Response to Reviewers.docx [file pone.0312797.s012.docx]

**Dear Editor, Dear Reviewer,**

We are grateful for the careful and constructive assessment of our manuscript. We appreciate the opportunity to revise and resubmit it. Please find our responses to the comments below.

Sincerely,

Prof. Marcin Waligora

Jagiellonian University Medical College

Krakow, Poland

**Response to Journal Requirements**

We updated the formatting as requested.

**Responses to the comments made by Omid Beiki**

**Introduction:**

- **Have you found any previous study on seamless trials in other fields than oncology? Please indicate it in introduction.**

**Response:**

Thank you for this comment. We added the following part to our manuscript:

*Clinical trials using the "seamless design" methodology are used across a range of medical disciplines, including for example cardiology, pulmonology, vaccine development [1, 11, 12]. Seamless design was also used in clinical trials conducted during the global pandemic caused by the SARS-CoV-2 virus [13, 14]. Nevertheless, the increasing significance of seamless design is particularly evident in the context of oncology research [4, 5, 15, 16].*

- **Lines 50-53: You have provided 9 references for one sentence. While they seem relevant to the sentence you have written, I prefer you to limit them to only the most relevant ones to the topic of study. Please consider those that you can later use in your discussion section.**

**Response:**

We are grateful for your feedback and have reduced the number of references to two.

**Methods:**

- **What was the age definition for adults and pediatrics?**

**Response:**

The categorization was adopted directly from the ClinicalTrials.gov registry. Each clinical trial record contains information regarding the age range of individuals who are eligible to participate in the study. This information is accessible via the “Participation criteria”. The definition of "age or age group" in the registry is as follows:

*A type of eligibility criteria that indicates the age a person must be to participate in a clinical study. This may be indicated by a specific age or the following age groups:*

*The age groups are:*

*- Child (birth-17)*

*- Adult (18-64)*

*- Older Adult (65+)*

We chose "Pediatrics" for the "Child" category or age under 18 (if the specific age range was reported). And "Adult" for the "Adult" and "Older Adult" categories and age over 18.

- **Could you please justify why you have selected 24 months for your analyses? Why not 12 months or 36 months? Considering the time to report results provided in table SB, I prefer to use 30 or 36 months to cover at least 75% of trials.**

**Response:**

Thank you for this comment. We added the justification to our manuscript (see Statistical analysis and tools).

*We chose 24 months for our analysis with reference to the World Health Organization's “Joint statement on public disclosure of results from clinical trials” [21]. This statement indicates that the primary results of a clinical trial should be reported in a registry within 12 months and published in a peer-reviewed journal within 12 months. Given the expected limited number of clinical trials that had reported results within the 12-month window, we decided to perform our statistical analysis in the 24-month window*

**Results:**

- **Table 1: I am not sure why you have reported percentages of second column as the ratio to all trials not trials with results. Please either justify the reason or provide the percentage to trails with results for each characteristic. As it is a descriptive table, I prefer to have the percentage to trails with results for each characteristic.**

**Response:**

Thank you for bringing this to our attention. We agree that the manner of presentation was misleading. Considering this comment, we have moved the data for characteristics of “Trials with results reported” from Table 1 to Table 2 and included a description of the percentage calculation in the footnote.

We also modified the title of Table 2 from *Comparison of clinical trials with results reported within 24 months* to: *A comparative analysis of results reporting rates based on the characteristics of the trials*

- **Table 2: It seems you calculated percentage based on all trials. If so, please specify it. Please specify the statistical test and the comparison group for P-value as well.**

**Response:**

Thank you for this comment. We added the following description to table footnote:

*^a^Percentages are calculated as the ratio of clinical trials with results to all trials within each trial characteristic category. For example, the reported percentage of 10.7% for Phase 1 trials in the "Trials with results reported within 24 months" column refers to the ratio of the number of Phase 1 trials with results reported within 24 months (60 trials) to the number of all Phase 1 trials (562 trials) in the cohort.*

*We used the chi-square or exact Fisher test to examine differences in results reporting rate by trial characteristics. P-value less than 0.05 was considered statistically significant.*

- **Table 3: Please specify all variables that were included in the model in table footnote. You should also specify CI and OR in table footnote same as US.**

**Response:**

We have added the expansion of the abbreviations OR and CI to the footnote as requested. In addition, we provided the following explanation:

*In the multivariable model, the characteristics that were statistically significant in the univariable analysis (see Table 2) were included and the backward elimination method was applied (P<0.1).*

**Responses to the comments made by Reviewer #1**:

**Abstract:**

- **In the Methods section, please correct "completed between" to "primary completion date" (PCD) to avoid confusion, as there is an important distinction between these terms. Additionally, when describing trials in the registry, it would be clearer to specify "reported as completed (PCD)" since trial status can vary across different registries. This clarification ensures that readers understand the definition being used.**

**Response:**

Thank you for bringing this to our attention. The suggested changes have been incorporated into the manuscript.

**Introduction:**

- **I would describe ClinicalTrials.gov as a relational database rather than just a tool. This distinction is critical for understanding the database's functionalities, such as querying and data extraction. However, consider this comment low priority.**

**Response:**

Thank you for pointing this. We have changed the term "tool" to "relational database" and added an appropriate reference.

**Search Strategy, Extraction form and Reproducibility:**

- **For reproducibility in generating the sample, please provide the exact search string used to identify relevant cancer trials, as mentioned in your protocol. It's important to note that using the term "cancer" may exclude some trials that are still relevant but coded under "neoplasm." While ClinicalTrials.gov searches for synonyms using MeSH terms, certain cancer trials could still be missed.**

**Response:**

Thank you. Now, we mention the exact search string in our manuscript.

*We used the following search string to generate the sample: Completed Studies | Interventional Studies | Cancer | Phase 1, 2 | Primary completion from 01/01/2016 to 12/31/2020. See more details about search strategy in S2 Table.*

We also added additional explanation in the limitation section:

*However, we acknowledge that our search strategy has some limitations. We used the term "cancer" to identify cancer clinical trials. We admit that this may have limited our sample with trials that used other terms e.g. neoplasm. Additionally, the ClinicalTrials.gov database underwent an interface change during the course of our study that affected both the description of certain elements in the clinical trial records and the functionality of the advanced clinical trial search tools.*

- **It would be better to attach the extraction form with the variables used. You can include it as a supplementary file, making it accessible as a downloadable file in the supplementary materials.**

**Response:**

We have attached the extraction form with the variables used as a supplementary file (see S1 File).

**Table 1 Characteristics:**

- **For better readability, the characteristics in Table 1 should be arranged in descending order of percentages. Additionally, please mention the inter-rater reliability (IRR) in the assessment of the extracted data. Consider this comment low priority.**

**Response:**

Thank you for bringing this to our attention. We have made the suggested changes to Table 1. Additionally, we have moved the column with the characteristics of trials with results reported to Table 2 for improved readability.

We have also performed the calculation of the inter-rater reliability (IRR) for the trial selection stage and added the following sentence to our manuscript:

The calculated the inter-rater agreement (Cohen's kappa) between the reviewers during the trial selection was 0.78 (95% CI: 0.75-0.80).

**Cross-Sectional Study Insights:**

- **In cross-sectional studies like this one, the extraction form and the data extracted can be crucial. The study's descriptive analysis should consider the representativeness of the sample, noting that a large proportion of the trials were conducted in North America. This regional concentration may limit the generalizability of the findings, so the results should be interpreted with caution. While the study discusses the results of seamless cancer clinical trials, it would have been more insightful to highlight how seamless trials differ from non-seamless trials in result reporting and characteristics.**

**Response:**

Thank you for bringing this to our attention.

In light of your feedback, we have acknowledged the regional limitation of our results.

*A significant number of the trials included in our analysis were conducted in North America. This regional concentration may limit the generalizability of the findings.*

To provide context for comparison, we have added the following sentences to our discussion:

*In comparison, another study that evaluated the reporting of results among all interventional oncology trials registered on ClinicalTrials.gov from 2007 through 2017 indicates that the results were posted on ClinicalTrials.gov for 22.9% (95% CI, 22.2%-23.7%) of the trials [23].*

**Overall Assessment:**

- **Thank you for the opportunity to review this article. My experience in evaluating data from trial registries and developing tools for assessing research practices in biomedical research gives me an appreciation for the importance of this study. However, I must emphasize, as the authors did, the limitations inherent in relying on registry data, particularly regarding trial registration and timely updates. Additionally, the phenomenon of cross-registration or registering the same trial in different registries, especially for non-North American trials, could mean that results might be reported elsewhere, potentially skewing the numbers. Given the North American bias in the sample, there is a regional limitation that should be acknowledged. Since no direct comparison between seamless and other trial designs was made within the sample, the study's primary value lies in its search strategy and extraction form. The meticulous approach to data extraction is commendable, and the authors' efforts to highlight the importance of presenting results for seamless trials, taking into account staging and cohort considerations, is noted.**

**Response:**

We would like to express our gratitude for the evaluation provided and the helpful comments that assisted us in making the necessary corrections to the manuscript. The responses and comments above illustrate the modifications that have been implemented. We hope this aligns with the feedback. We also mentioned in our limitations about potential bias of publication results in other clinical trials registries:

*Results may have been reported elsewhere, potentially biasing the numbers. Further analyses should be conducted to assess the availability of results in other sources, including journal publications or other clinical trial registries (due to the phenomenon of cross-registration of the same trial in different registries, especially for non-North American trials).*
